# Successful revision surgery after postsurgical pyoderma gangrenosum following reduction mammaplasty: A case report

**DOI:** 10.1016/j.jpra.2026.06.012

**Published:** 2026-06-29

**Authors:** Lindsay A. Tao, Carolyn Cafro, Micaela Rosser, Elsy Compres, Anna K. Haemel, Esther A. Kim

**Affiliations:** a University of California, San Francisco, Department of Surgery, Division of Plastic and Reconstructive Surgery, San Francisco, CA, USA; b University of Rochester School of Medicine and Dentistry, Rochester, NY, USA; c University of California, San Francisco, Department of Dermatology, San Francisco, CA, USA

**Keywords:** Pyoderma gangrenosum, Post-surgical pyoderma gangrenosum, Reduction mammaplasty. breast surgery complications, Reoperation, Case report

## Abstract

**Background:**

Postsurgical pyoderma gangrenosum (PSPG) is a rare neutrophilic dermatosis that occurs following surgical procedures and frequently mimics postoperative infection. Misdiagnosis may lead to repeated surgical debridement and disease progression due to pathergy. Reports describing safe reoperation following PSPG remain limited, creating uncertainty regarding subsequent reconstructive procedures.

**Case:**

We report the case of an 18-year-old woman with well-controlled perinatal HIV who developed PSPG following bilateral reduction mammaplasty. On postoperative day (POD) 2 she developed refractory fevers and blisters near the vertical incision. By POD 6, ulceration developed on the left breast T-junction, prompting hospital admission. Dermatologic consultation and biopsy confirmed neutrophilic dermatosis consistent with pyoderma gangrenosum (PG). Multidisciplinary management involving dermatology, infectious disease, and plastic surgery led to initiation of systemic corticosteroids and immunomodulatory therapy, resulting in progressive clinical improvement and complete wound healing by eight months. After sustained disease remission and negative pathergy testing, the patient underwent staged reconstructive revision to address hypertrophic scarring and contour deformity. Revision mastopexy and autologous fat grafting were performed while the patient remained on biologic immunomodulatory therapy, with operative techniques modified to minimize cutaneous trauma. The patient healed without recurrence of PG.

**Conclusion:**

Postsurgical pyoderma gangrenosum presents a unique challenge for reconstructive surgeons because surgical trauma may trigger disease progression or recurrence through pathergy. This case demonstrates that revision surgery may be feasible after PG remission when disease control is sustained and perioperative management is coordinated with dermatology. Continued immunomodulatory therapy and surgical techniques that minimize cutaneous trauma may help mitigate recurrence risk and facilitate safe reconstructive revision.

## Introduction

Pyoderma gangrenosum (PG) is a rare ulcerative neutrophilic dermatosis characterized by rapidly progressive cutaneous ulceration and inflammatory tissue destruction.^1^ PG may occur spontaneously or in association with systemic conditions including inflammatory bowel disease and rheumatologic disorders.^2^

Postsurgical pyoderma gangrenosum (PSPG), characterized by the development of PG-lesions within surgical sites, represents a particularly challenging clinical entity.^3^ Early manifestations frequently resemble surgical site infection, presenting with fever, leukocytosis, wound drainage, and progressive ulceration. Surgical trauma exacerbates disease through pathergy, wherein minor trauma provokes an exaggerated inflammatory response. Thus, repeated surgical debridement may worsen tissue destruction and delay appropriate therapy.^3^ Prompt recognition and multidisciplinary management are therefore critical to initiate immunosuppressive therapy and prevent unnecessary debridement.

Although PSPG has been described following breast surgery, guidance regarding subsequent reconstructive procedures remains limited.^4^ Because surgical trauma may trigger recurrence, many surgeons remain hesitant to perform additional procedures after disease resolution. Existing literature has focused primarily on initial recognition and management of acute PSPG, resulting in a paucity of evidence to guide the timing and perioperative management of revision surgery. We present a case of PSPG following bilateral reduction mammaplasty that subsequently underwent successful staged reconstructive revision after sustained disease remission. This case is distinguished by the use of a structured perioperative strategy incorporating negative pathergy testing, continuation of immunomodulatory therapy, and multidisciplinary coordination with dermatology. By detailing these considerations, we aim to highlight practical insights into the safe pursuit of revision procedures following PSPG.

## Case presentation

An 18-year-old African American woman with well-controlled perinatal HIV (undetectable viral load on antiretroviral therapy, CD4 834) presented with symptomatic macromastia. She had no prior surgical history, and preoperative evaluation was unremarkable. Bilateral breast reduction was performed using a Wise pattern skin excision with superomedial pedicle. The procedure was uncomplicated, and the patient was discharged the same day.

**Fig. 1:**
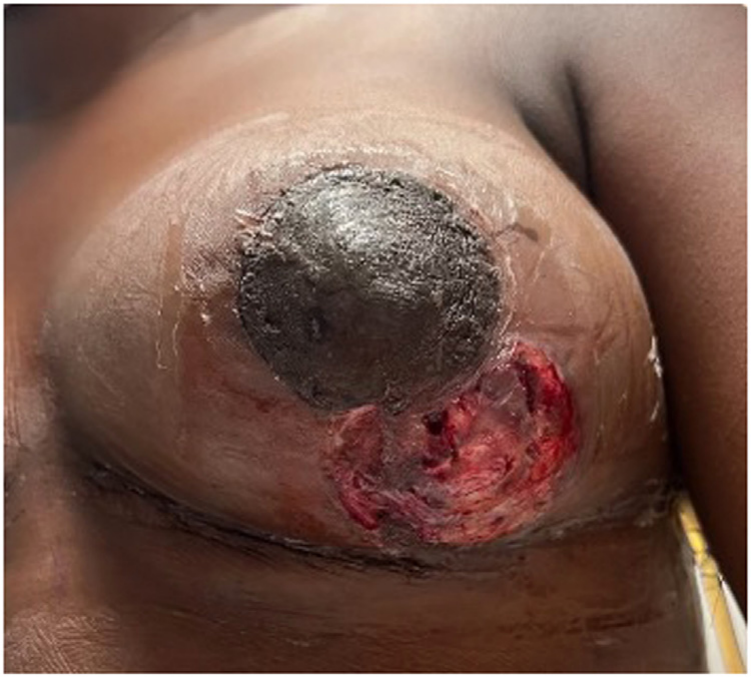
Postoperative day 6 following breast reduction. Left breast with large ulceration with focally violaeous borders and early undermining.

**Fig. 2:**
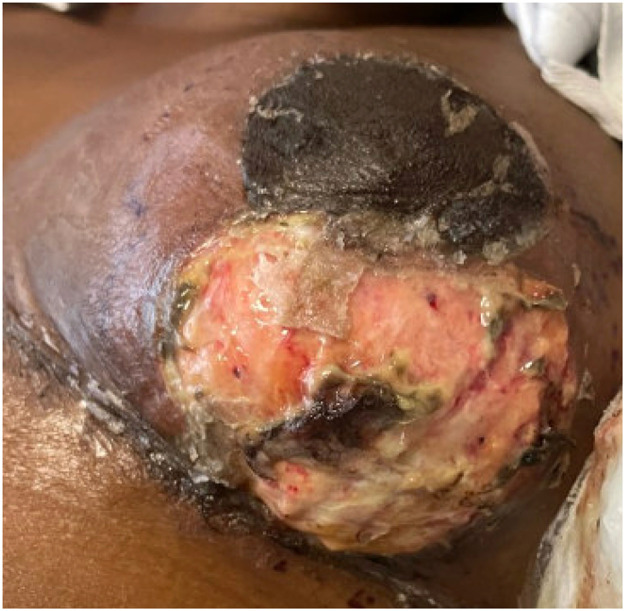
Postoperative day 9 following breast reduction. Left breast with progression of PG related ulcer with inflammatory borders and copious fibrinous debris prior to disease control with immunomodulators.

On postoperative day (POD) 2, she developed fevers to 104°F despite acetaminophen and was empirically started on trimethoprim-sulfamethoxazole given her HIV history, though opportunistic infection was unlikely. By POD 4, she developed blisters at the left breast with persistent fever and neutrophilic-predominant leukocytosis (14.6 K). On POD 6, the lesion progressed to a large ulceration with violaceous undermined borders, prompting hospital admission for suspected PG (Fig. 1). She later developed new ulcerations on the right breast with progression of the left-sided lesion (Fig. 2, Supplementary Figure 1).

**Fig. 3:**
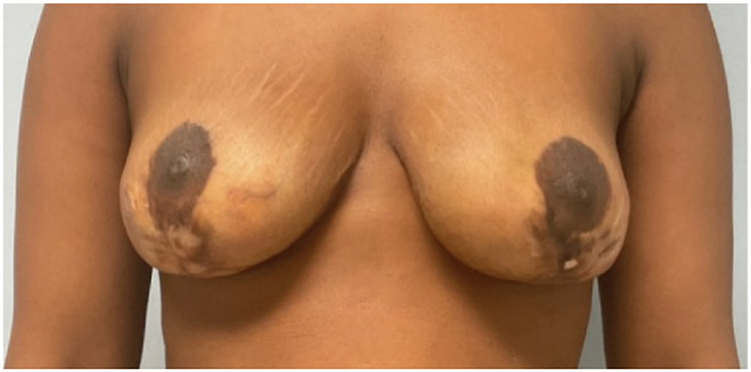
8 month postoperatively after breast reduction. Complete healing of PG ulcerations with post- inflammatory hyperpigmentation and hypertrophic scars.

Multidisciplinary evaluation involving dermatology, infectious disease, and plastic surgery was initiated. Punch biopsy demonstrated neutrophilic dermatosis consistent with PSPG, further supported as infectious workup including wound cultures remained negative. Initial dermatologic management included gentle wound care and topical anti-inflammatory therapy with clobetasol and tacrolimus, and mupirocin while infection remained under consideration. Because the lesions continued to progress, intralesional triamcinolone (10 mg/mL) was administered to the ulcer margins and systemic immunosuppression was escalated to pulse-dose intravenous methylprednisolone (500 mg daily for three days), followed by transition to high-dose oral prednisone (80 mg daily with gradual taper). This decreased inflammation and stabilized ulcer progression. Steroid-sparing biologic therapy was subsequently introduced outpatient, consisting of infliximab induction followed by adalimumab maintenance (80 mg weekly) after infliximab was poorly tolerated. Wound care continued with topical tacrolimus, clobetasol, silver sulfadiazine, and soft silicone atraumatic dressings to minimize cutaneous trauma and support epithelialization. The patient stabilized with no progression of ulcerations within one month, and complete wound healing by eight months (Fig. 3).

After sustained remission on adalimumab and negative pathergy testing, reconstructive revision was pursued to address hypertrophic scarring and contour deformity. The patient was intentionally maintained on adalimumab because of her desire for future revision procedures; otherwise, systemic therapy would have been tapered within several months of full healing of the initial wounds. A staged approach was planned, with autologous fat grafting to improve contour, followed by revision mastopexy to address scars from secondary wound healing.

**Fig. 4:**
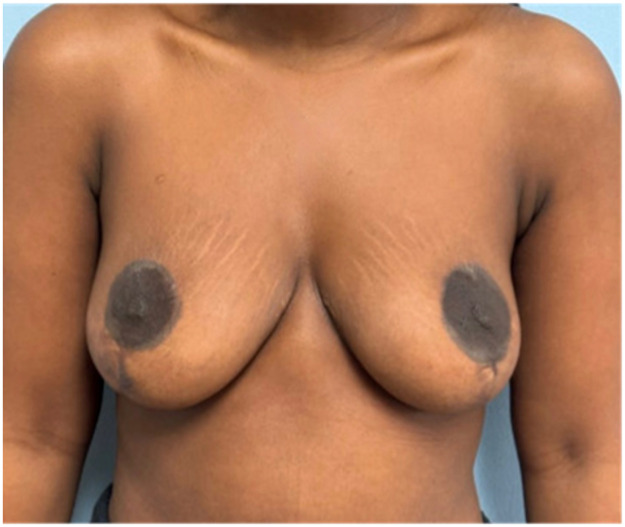
10 months postoperative follow up after secondary revision surgery.

During the revision procedure, force-modulating tissue bridges (Brijjit) were applied to offload tension and reduce wound dehiscence risk, given dermal thinning from multiple prior intralesional triamcinolone injections. Prednisone 40 mg was initiated postoperatively and subsequently tapered. The patient later underwent nipple-areola tattooing to improve asymmetry. She healed without recurrence of PG and achieved a satisfactory reconstructive outcome (Fig. 4).

## Discussion

PSPG remains a challenging diagnosis for surgeons because early manifestations frequently resemble surgical site infection. Fever, leukocytosis, and progressive wound breakdown often prompt empiric antibiotic therapy or surgical debridement; however, surgical trauma may exacerbate PG through pathergy, worsening tissue destruction.^5^ Recognition may be particularly difficult in patients with melanotic skin, where classic features such as erythema and violaceous borders may be less apparent. In this case, early clinical suspicion and multidisciplinary decision-making were critical to diagnosing and guiding management. Although initial fever, leukocytosis, and wound drainage raised concern for infection, the rapid progression, lack of response to antibiotics, and bilateral ulcerations at surgical sites raised suspicion for PG, prompting dermatologic evaluation.

Management of PG centers on early recognition and prompt initiation of immunosuppressive therapy. First-line systemic therapy typically includes corticosteroids or cyclosporine to rapidly suppress neutrophilic inflammation and halt disease progression. Now, biologic agents targeting tumor necrosis factor-α, including infliximab and adalimumab, have demonstrated efficacy and are widely used in moderate-to-severe PG.^1^^,^^6^ Adjunctive therapies include intralesional corticosteroids, topical immunomodulators, and meticulous wound care to minimize mechanical trauma. Multidisciplinary management involving dermatology is critical to guide therapy and monitor disease control prior to further surgery.

Beyond the diagnostic challenges of PSPG, it remains unclear whether patients with PSPG can safely undergo subsequent surgical procedures.^4^^,^^7^ Because of the risk of pathergy-induced recurrence, reconstructive revision procedures are often deferred despite persistent functional or aesthetic sequelae.

Published reports describing reoperation after PSPG are scarce. One case report described successful reoperation with gender-affirming top surgery in a patient who initially developed PG after a breast reduction, demonstrating that surgical revision may be feasible in selected cases.^8^ Here, perioperative immunosuppression with prednisone and cyclosporine was used to mitigate recurrence risk. In contrast, Caddia et al. described recurrence of PG following revision surgery after reduction mammaplasty. Although they noted potential benefits of prophylactic immunomodulation, concerns included impaired wound healing and risk of dehiscence. Instead, they emphasized close postoperative monitoring and corticosteroid therapy if recurrence occurs.^9^ In both reports, reoperation was attempted only after initial disease management, although the degree of disease quiescence at the time of surgery was not uniformly characterized. Timing relative to disease control may represent a critical determinant; reoperation may be safer after sustained remission, rather than early or incompletely controlled disease, where ongoing inflammation may predispose to pathergy and recurrence. These reports demonstrate that reoperation after PSPG is possible, but their variable outcomes highlight the uncertainty surrounding optimal perioperative management in patients with prior PG.

Our case contributes to this limited body of literature by delineating a successful approach to staged reconstructive revision following PSPG while the patient remained on maintenance biologic therapy. Several factors likely contributed to this favorable outcome, including prolonged disease stability, negative pathergy testing, and continued biologic therapy with brief perioperative corticosteroid support. While a single case cannot establish causality, these considerations may represent important elements of a perioperative strategy aimed at reducing recurrence risk. In our practice, we recommend maintaining patients on biologic therapy such as adalimumab through the perioperative period and for approximately four to six weeks following surgery to reduce the risk of PSPG recurrence. Use of perioperative steroids remains debated, given potential deleterious effects on wound healing. Tension-offloading devices such as Brijjit may address concern for increased risk of wound dehiscence in patients with compromised dermal integrity.^10^ Additionally, trauma-minimizing operative strategies, namely favoring subcuticular closures, minimizing suture bulk, and limiting epidermal suture tracking may help mitigate the risk of pathergy-associated recurrence.

These findings suggest that reoperation after PSPG may be feasible when sustained disease remission has been achieved and multidisciplinary coordination maintained. Careful collaboration with dermatology to ensure ongoing disease control, along with thoughtful perioperative management and surgical technique, may allow reconstructive revision with favorable surgical and aesthetic outcomes. For patients left with significant aesthetic consequences following PSPG remission, safe surgical reconstruction may represent an important opportunity to restore form and improve long-term outcomes.

## Ethical approval

Written informed consent was obtained from the patient for publication of this case report. The report was prepared in accordance with the Declaration of Helsinki.

## Funding sources

None.

## Declaration of competing interest

None.

## Appendix Supplementary materials

Image, application 1

Image, image 2 dummy alt text
