# Supplementary material for: Successful revision surgery after postsurgical pyoderma gangrenosum following reduction mammaplasty: A case report
Source: JPRAS Open. 2026 Jun 29;51:357–62. doi: 10.1016/j.jpra.2026.06.012 (PMC13427583; doi:10.1016/j.jpra.2026.06.012)
Supplement: Supplementary file 1 [file mmc1.docx]

**Supplemental Figures:**

**Supplemental Figure 1.** Postoperative day 9 following breast reduction. Right breast with developed PG-related ulcer with inflammatory borders and copious fibrinous debris prior to disease control with immunomodulators.
