# Supplementary figures and images for: Successful revision surgery after postsurgical pyoderma gangrenosum following reduction mammaplasty: A case report
Source: JPRAS Open. 2026 Jun 29;51:357–62. doi: 10.1016/j.jpra.2026.06.012 (PMC13427583; doi:10.1016/j.jpra.2026.06.012)

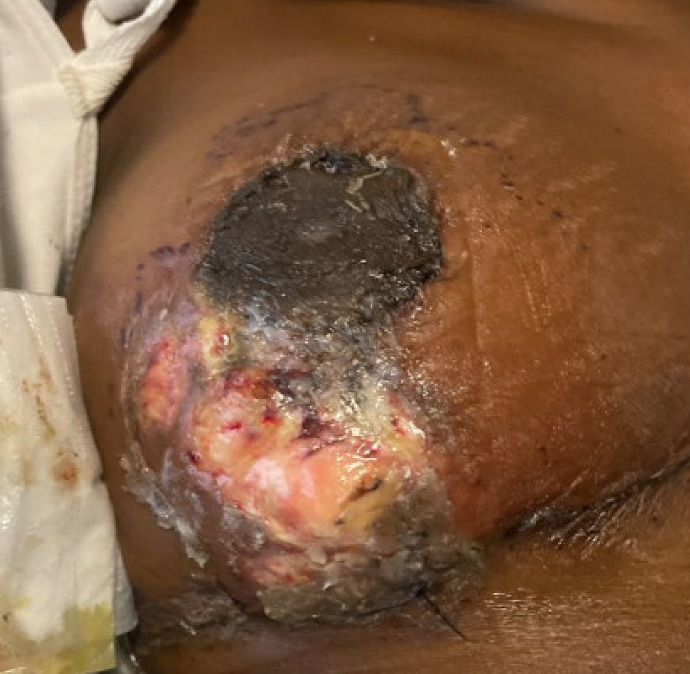

Supplement: Supplementary file 2 [file mmc2.jpg]
